# Kinematical, Structural and Mechanical Adaptations to Desiccation in Poikilohydric *Ramonda myconi* (Gesneriaceae)

**DOI:** 10.3389/fpls.2018.01701

**Published:** 2018-11-20

**Authors:** Tim Kampowski, Sven Demandt, Simon Poppinga, Thomas Speck

**Affiliations:** ^1^Plant Biomechanics Group Freiburg (PBG), Botanic Garden, University of Freiburg, Freiburg im Breisgau, Germany; ^2^Freiburg Materials Research Center (FMF), University of Freiburg, Freiburg im Breisgau, Germany

**Keywords:** desiccation tolerance, functional morphology, leaf folding kinematics, plant biomechanics, resurrection plants

## Abstract

Resurrection plants have fascinated scientists since centuries as they can fully recover from cellular water contents below 10%, concomitantly showing remarkable leaf folding motions. While physiological adaptations have been meticulously investigated, the understanding of structural and mechanical adaptations of this phenomenon is scarce. Using imaging and bending techniques during dehydration-rehydration experiments, morphological, anatomical, and biomechanical properties of desiccation-tolerant *Ramonda myconi* are examined, and selected structural adaptations are compared to those of homoiohydrous *Monophyllaea horsfieldii* (both Gesneriaceae). At low water availability, intact and cut-off *R. myconi* leaves undergo considerable morphological alterations, which are fully and repeatedly reversible upon rehydration. Furthermore, their petioles show a triphasic mechanical behavior having a turgor-based structural stability at high (Phase 1), a flexible mechanically state at intermediate (Phase 2) and a material-based stability at low water contents (Phase 3). Lastly, manipulation experiments with cut-off plant parts revealed that both the shape alterations of individual structures, as well as, the general leaf kinematics largely rely on passive swelling and shrinking processes. Taken together, *R. myconi* possesses structural and mechanical adaptations to desiccation (in addition to physiological adaptations), which may mainly be passively driven by its water status influenced by the water fluctuations in its surroundings.

## Introduction

About 400 million years ago plants left water and conquered land. Since then, land dwelling plants have been challenged by an exposure to a dry atmosphere, as well as, to associated water stresses. As a consequence, they have evolved various adaptations to evade and minimize water-deficit stresses and the related negative effects (Bateman et al., 1998; Lüttge et al., 2011). Such adaptations include the production of drought-tolerant seeds and spores, the development of a water-proof cuticle with stomata and water-storing tissues, or the exploitation of deep water sources with extensive root systems (Bewley, 1979). One of the most extreme adaptations to drought is termed desiccation tolerance (DT) and can be observed in poikilohydric plant species, which include the resurrection plants in angiosperms (Lüttge et al., 2011). In contrast to drought describing the condition of low environmental water availability, desiccation is defined as the state, in which a plant has lost most of its cellular water, resulting in an absolute water content of 0.1 g H_2_O g^−1^ dry mass (which is equivalent to air-dryness at 50% relative humidity (RH) and 20°C with a corresponding water potential ≤ −100 MPa) (Iljin, 1957; Vertucci and Farrant, 1995). Therefore, desiccation-tolerant organisms are always drought-tolerant, with the opposite not necessarily being true (Moore et al., 2008).

Poikilohydric species can be found in non-vascular (e.g., algae, bryophytes) and vascular plants (e.g., ferns and allies, angiosperms) (Proctor et al., 1998; Oliver et al., 2000). Moreover, one discerns in DT plants homoiochlorophyllous (HDT) and poikilochlorophyllous (PDT) species. While HDT plants generally keep their chlorophyll during severe droughts, PDT plants reversibly degrade their chloroplasts to desiccoplasts, thereby losing their chlorophyll completely (Tuba et al., 1994; Sherwin and Farrant, 1998; Farrant et al., 2007). Thus, HDT plants are primarily adapted to survive frequently occurring short and moderate dry periods, whereas PDT plants, although being less flexible due to time-consuming metabolic rearrangements, are able to survive prolonged phases of severe dehydration.

Both HDT and PDT species may have morphological traits or may perform macroscopic plant movements, which minimize the total amount of transpiration and optimize the plant's surface to volume ratio, e.g., by possessing small hairy leaves, a small habitus, and leaf folding or rolling in movements. All poikilohydric plants possess adaptations contributing to the replacement or the reduction of the cellular water content (Gaff, 1971; Farrant, 2000)_._ A concert of well-coordinated yet complex interactions of physiological, biochemical, and structural adaptations prevents such plants from permanent dehydration- and rehydration-induced damages. On a physiological and biochemical level, for the preservation of the cell integrity three mechanisms have been commonly observed and referenced to: (1) detoxification of reactive oxygen species (ROS), (2) synthesis of late embryogenesis abundant (LEAs) and small heat shock proteins (sHSPs), and (3) the concentration of sugars (e.g., sucrose or trehalose) (Alamillo et al., 1995; Farrant, 2000; Kranner et al., 2002). So far, structural adaptations of HDT and PDT species have not been investigated in detail, and our current knowledge is mainly based on the afore-mentioned macroscopic movements, as well as, on concomitant ultrastructural cell wall folding (Vicré et al., 1999, 2004; Farrant, 2000; Moore et al., 2006).

The HDT gesneriad *Ramonda myconi* grows on rocky substrates in the Pyrenees and regularly faces extreme temperatures and high insulation due to high altitudes and sparse vegetation, periodically occurring droughts due to low water storage capacities of the substrates, as well as, high exposure to external mechanical loads caused by wind, rain and rock fall (Stevanović et al., 1997; Drazić et al., 1999; Riba et al., 2002; Jovanović et al., 2011; Rakić et al., 2014a,b). Like other DT Gesneriaceae, *R. myconi* shows both specialized physiological and structural properties to survive under such harsh conditions (Navari-Izzo et al., 2000; Gechev et al., 2013).

Here, we focus on structural and mechanical adaptations of *R. myconi* by analyzing its kinematical, morphological, anatomical, and biomechanical properties. First, we performed qualitative dehydration-rehydration experiments (DREs) on intact *R. myconi* plants to understand how their general morphology is affected by changes in water availability. Afterwards, we related water content alterations to the respective plant's tissue architecture by combining DREs with anatomical analyses. Similarly, we explored how plant stability varies with changes in water content. Therefore, we investigated the biomechanical properties of *R. myconi* petioles using bending tests during the DREs. Additionally, we investigated if *R. myconi*'s adaptations to desiccation also include structural means by using a set of manipulation experiments. Finally, we evaluate how the structural and biomechanical traits contribute to the desiccation tolerance of *R. myconi*.

## Materials and methods

### Plant material and dehydration-rehydration experiments (DREs)

*Ramonda myconi* (L.) Rchb. (Gesneriaceae) was purchased from Kaiserstühler Staudenhof (Menton GdbR, Eichstetten, Germany) and cultivated in a cold greenhouse (11°C, 64% relative humidity, RH) of the Botanic Garden of the University of Freiburg. In-between test periods, all plants were kept at well-watered conditions. The DREs followed a “generalized” setup comprising 12-days dehydration and 5-days rehydration. At the end of day 12, the beginning of the rehydration period, all plants were simultaneously irrigated to field capacity. During each DRE, increasingly dehydrated samples were collected at days 0, 3, 4, 5, 6, 7, 10, 11, and 12, whereas progressively rehydrated samples were gathered at days 12.5, 13, 13.5, 14, and 17 (test intervals). Only mature plants showing no visible leaf or petiole damage were subjected to experiments. For additional comparative measurements, *Monophyllaea horsfieldii* R.Br. plants (accession-no.: 13885; IPEN: xx-0-BONN-13885; herbarium-no.: 1727), were cultivated according to (Kampowski et al., 2017).

### Relative water content (RWC) measurements

Circular discs with a diameter of 3 mm were punched out of the laminae using an arc punch (Matador, Remscheid, Germany) to gather leaf samples of *R. myconi*, whereas petioles were cut with razor blades each into four equally long sections, with the actual sample lengths depending on the total length of the respective petiole. Each sample was weighed (UMT2 high-precision balance, Mettler-Toledo, Greifensee, Swiss) immediately after excision to determine its fresh weight (FW). Afterwards, the sample was dark incubated in distilled water for 2 h at RT to reach its turgescent state and measure its turgescent weight (TW) having removed all of the excess water in advance. Lastly, the sample was dried for 24 h at 80°C in a heating cabinet (TK/L4250, Ehret, Emmendingen, Germany) and weighed a third time to determine its dry weight (DW). A detailed description for the calculation of RWCs from the respective fresh, turgescent and dry weights can be found in Kampowski et al. (2017). To investigate location-specificity, RWCs from four lamina sample pairs (one left and one right sample per pair within a basipetal progression of the pairs) and four petiole samples (basipetal progression of the petiole samples) were measured and compared at each test interval during a generalized DRE (three replications).

### Light microscopy

Transverse and longitudinal petiole and lamina thin sections of *R. myconi* leaves were stained with 0.05% aqueous toluidine-blue (TB) or with 3% aqueous acridine-orange (AO) after embedding in a methacrylate-based resin (Technovit 7200, Kulzer, Wehrheim, Germany) according to the manufacturer specifications. The embedded samples were cut with a thickness of 5 μm using a custom-build rotary microtome (Technical Workshop, Institute of Biology II/III, University of Freiburg, Germany), stained with TB or AO staining solutions for ~ 30 s, washed with distilled water and permanently mounted on microscope slides using Entellan® mounting medium (Merck KGaA, Darmstadt, Germany). We used two different microscopy settings according to the different staining procedures: (1) TB thin sections were examined using transmitted light, and (2) AO-stained thin sections were imaged under fluorescent light applying excitation and emission wavelengths of 494 nm and 518 nm, respectively. All images were acquired with a BX61 microscope (Olympus, Tokyo, Japan) equipped with a digital camera (DP71, Olympus, Tokyo, Japan) using the cellP software (v2.8, Olympus, Tokyo, Japan). Additionally, fresh cross-sections of petioles and laminae were stained with Phloroglucin-HCl to evaluate lignification patterns (see Figure S3).

### Water-dependent changes in morphology of *R. myconi*

#### Morphometry of petioles during DREs

Ten morphometric parameters were measured on both leaf lamina and petiole samples across all DREs with a caliper and assorted according to their respective RWCs: the length and width of the leaf lamina; the basal, median and apical thickness of the leaf lamina; the thickness of and between first order veins; and the length, height, and width of the petiole.

#### Leaf folding kinematics of whole plants during DRE

The leaf folding kinematics of a whole *R. myconi* plant was analyzed in a 20-days dehydration and 4-days rehydration setup at 21°C and 47% RH. The experiment was replicated four times and was the only one which did not follow the generalized DRE setup. Single images were acquired from lateral and top views using digital USB microscope cameras (Conrad, Hirschau, Germany), portable spotlights (Jet-Light ELH 38 IP 54, Hugo Brennenstuhl, Tübingen, Germany) and the freeware Yawcam (v0.5.0, by Magnus Lundvall) at frame capturing rates of 2 frames h^−1^ and 12 frames h^−1^ for dehydration and rehydration phases, respectively. All acquired images were converted to time-lapse videos (playback rate 30 fps) using ImageJ (v1.50b, U.S. National Institutes of Health, Bethesda, Maryland, USA) (see Video S1).

### Anatomy of *R. myconi* petioles during DREs

**Figure 1:**
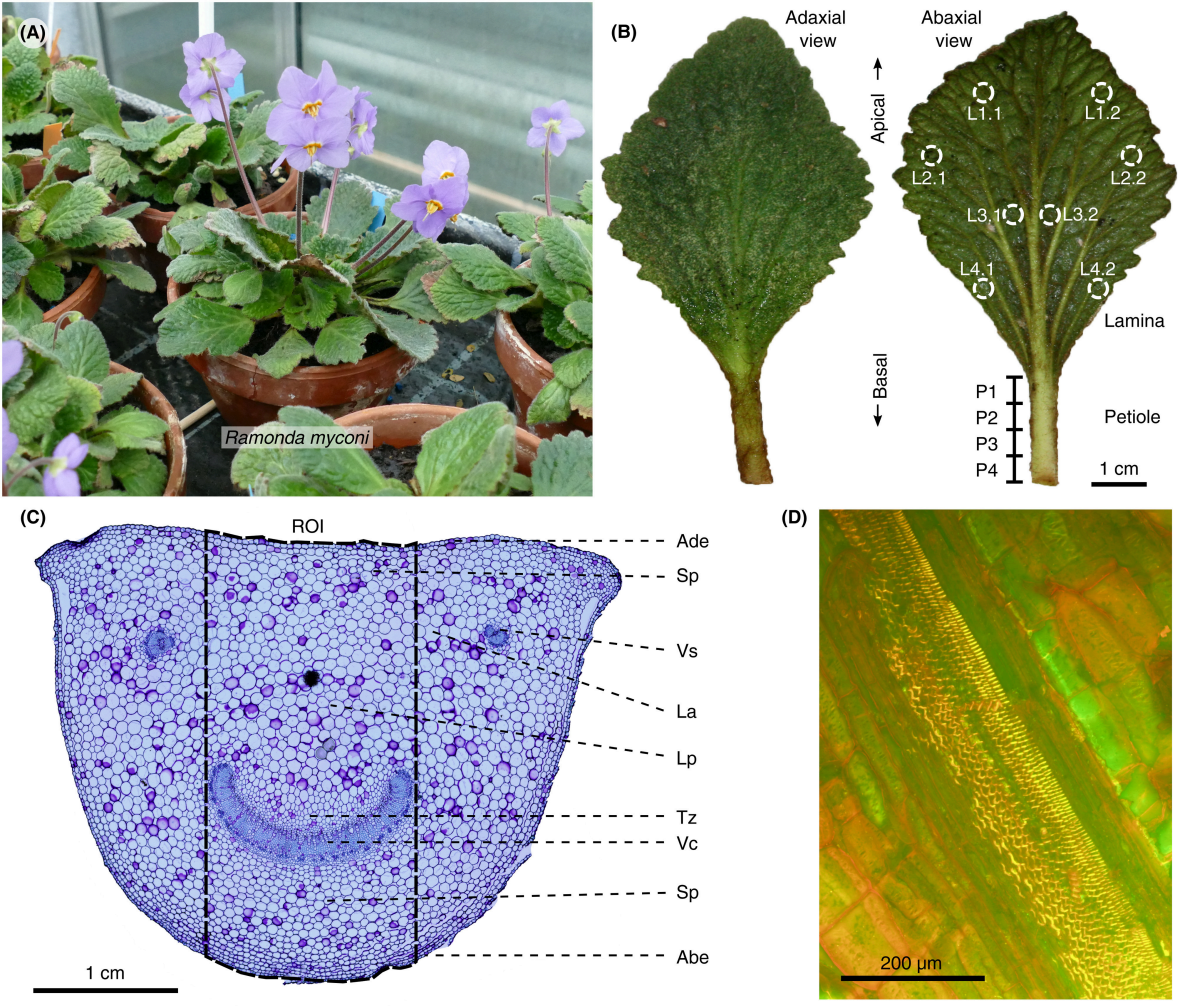
Habitus, leaf morphology and anatomy of *R. myconi*. **(A)** Cultivated flowering plants. **(B)** Adaxial and abaxial view of *R. myconi* leaves. Sampling areas for the location-specific RWC measurements are marked on the lamina and petiole of the right leaf. L1–L4: apical to basal lamina sampling areas [additional indices indicate locations left (1) or right (2) from the midrib]; P1–P4: apical to basal petiole sampling areas. **(C)** Cross-section of the petiole of *R. myconi* stained with TB. The image highlights all tissue types and the ROI used for the anatomical measurements in each thin section. ABE, abaxial epidermis; ADE, adaxial epidermis; LA, lacuna; LP, large-celled parenchyma (with pronounced lacunae); SP, small-celled parenchyma; TZ, transition zone between vascular and parenchymatous tissues; VC, vascular complex; VS, vascular strand. **(D)** Close-up of a longitudinal cut of the petiole showing the spiral thickenings of the xylem vessels (AO staining).

Fresh cross-sections (100 μm thickness) of *R. myconi* petioles were prepared using a hand microtome (MT.5503, Euromex Microscopen, Arnhem, Netherlands) and analyzed according to their tissue specific cell area changes at varying water contents. The plants (*N* = 10) were kept at 23°C and 64% RH throughout one generalized DRE (one replication). At every test interval, one petiole per plant was sampled and embedded in elder pith to facilitate the thin sectioning procedure with the hand microtome. All thin sections were immediately imaged with a stereo microscope (SXZ9 with DF PLAPO 1X^−2^ objective, Olympus, Tokyo, Japan) equipped with a digital camera (PL-D685C4, PixeLINK, Ottawa, Canada) using the software μ*Scope* (v20.1, IMT i-Solution Inc., Ottawa, Canada). Petiole remnants were used for standard RWC measurements. Only the thin sections of highest quality per petiole were digitally processed for the subsequent anatomical analyses (88 cuts in total). All images were digitally oriented and converted to grayscale using GIMP (v2.8.16, 2001-2016 the GIMP Team). Additionally, contrast and brightness levels were adjusted in order to facilitate the tissue specific cell area measurements in ImageJ. As region of interest (ROI), the area between the adaxial and abaxial cross-section borders and two parallel lines being tangents to the edges of the abaxially located curved vascular bundle complex was analyzed in all petiole sections (Figure 1C). Inside this ROI, the areas of ten randomly selected cells were quantified for each of the seven different tissues layers per cut.

### Petiolar biomechanics of *R. myconi* during DREs

One petiole per plant (*N* = 30) was tested in three-point bending measurements at every test interval during a generalized DRE (one replication). Prior to bending, each sampled leaf was measured morphometrically (see section Morphometry of Petioles During DREs) before the lamina was cut off and the petiole was placed onto custom-made specimen holders (Technical Workshop, Institute of Biology II/III, University of Freiburg, Germany) on the universal testing machine (Instron 4466-10 kN, with a retrofit kit to inspect-DC standard, Hegewald and Peschke Mess- und Prüftechnik GmbH, Nossen, Germany). All bending measurements were conducted with a 2.5 N force sensor, a testing velocity of 1 mm min^−1^ and a total displacement of 1 mm per test. For each test, the spacing of the supports was maximized to achieve the highest possible length-to-diameter ratio, thereby minimizing the influence of shear forces (Vincent, 1992). If plants were not mechanically testable due to severe water-loss induced slackening, the measurements on such plants were continued at the next possible test interval in the further course of the DRE. Directly after bending, three petiolar cross-sections (from basal, median and apical origins) were prepared manually and imaged using a digital USB microscope camera (Conrad, Hirschau, Germany) and the software Yawcam. Subsequently, the images were oriented and cut out digitally using GIMP in order to analyze their axial second moments of area (I_ax_) using ImageJ together with the BoneJ plug-in (v1.4.1) (Doube et al., 2010). Petiole remnants were used for standard RWC measurements. Finally, the structural bending elastic modulus was calculated in GNU R (v.3.2.5) using Equation 1 (R Core Team, 2016).

(1) $$\begin{array}{r} E\left(MPa\right)=\frac{L^{3}}{\left(48\cdot b\cdot I_{ax}\right)} \end{array}$$

with *b* being the slope of the displacement-force diagram, *I*_*ax*_ being the axial second moment of area from the BoneJ analyses and *L* being the spacing length between the supporting points. However, since the petiole diameters strongly varied due to RWC changes during the DRE, it was impossible to maintain constant length-to-diameter ratios throughout the course of bending measurements. Therefore, the structural bending moduli were normalized to the median structural bending modulus of fully turgescent petioles.

### Assessment of structural adaptations to desiccation

Starting with a specific drying experiment, the general structural regeneration capacity of *R. myconi* petioles was compared to that of hypocotyl sections of the closely-related desiccation-intolerant *M. horsfieldii* (one replication). Particularly, petiole and hypocotyl samples (*N* = 10) were imaged (EOS 400D SLR camera, Canon, Tokyo, Japan) and weighed (UMT2 High-precision balance, Mettler-Toledo, Greifensee, Swiss) directly after excision to determine their fresh weights (FW). Subsequently, the samples were dried in a heating cabinet (TK/L4250, Ehret, Emmendingen, Germany) for 8 h at 80°C. Afterwards, all petiole and hypocotyl samples were submersed and dark-incubated in distilled water at RT for 24 h before the final imaging and weighing steps (rehydrated weights, RWs) were performed. Finally, the percentage recovery (PR) values of *R. myconi* and *M. horsfieldii* were quantified according to Equation 2.

(2) $$\begin{array}{r} \text{PR}\left(\%\right)=\frac{RW}{FW}.100 \end{array}$$

Furthermore, the folding and shrinking behavior of cut-off *R. myconi* leaves was analyzed in several specific DREs (one replication per type of manipulation experiment). Firstly, the folding and shrinking behavior of cut-off leaves was compared to the behavior observed for intact plants. The cut-off leaves were air-dried in an empty water tank and simultaneously imaged from lateral and top view using the digital USB microscope cameras, portable spotlights and the Yawcam freeware mentioned above at a frame capturing rate of 12 frames·h^−1^. After dehydration, the water tank was flooded with tap water for rehydration with identical imaging settings. One leaf went through three consecutive dehydration-rehydration cycles (cycle 1: 3-days dehydration and 3-days rehydration; cycle 2: 4-days dehydration and 4-days rehydration; cycle 3: 4-days dehydration and 3-days rehydration) to evaluate the reversibility of the folding and shrinking behavior. Finally, the acquired images were converted to time-lapse videos using ImageJ (see Videos S2–S3). Additionally, the role of the venation system in context of leaf folding and shrinking was analyzed in another water tank DRE setup testing leaves with excised laminae (see Video S4). Afterwards, the lamina parts and the venation system of one cut-off leaf were separated, clamped in-between transparent plastic petri dishes, and video captured to individually evaluate their contributions to the 2D motions occurring during dehydration and rehydration (see Video S5). Finally, freshly cut-off leaves were pinned with their laminae to a vertically positioned wooden plank and weighed with 1, 5, and 10 g mass objects attached to the respective petioles (**Figure 6T**). Both the shortening of the petioles, as well as, the resulting forces during shrinkage were determined qualitatively to gain first insights into the pulling forces required for the retraction of the leaves toward their vegetation point.

### Statistical analysis

Two-tailed statistical analyses were performed with the software GNU R v.3.2.5 including the additional packages *car* and *psych* (Fox and Weisberg, 2011; Revelle, 2015; R Core Team, 2016). Primarily, we analyzed our datasets using descriptive statistics after having checked the assumptions for normally distributed data (Shapiro-Wilk test) and homoscedasticity of the variances (Levene test) in advance. As average and dispersion characteristics, we either used mean and standard deviation for parametric or median and interquartile range for non-parametric data. Statistically significant differences between different points of time or RWC states during DREs were analyzed in case of the mechanical and anatomical investigations performing Kruskal-Wallis tests due to non-parametric data (only groups with *N* > 5 were included in these statistical analyses; for groups with *N* < 5 only the measured values are given in the respective figures). Here, pairwise Wilcoxon rank sum tests with adjusted *p*-values (using Holm's method) were applied *post-hoc*. An alpha level of 5% was used for all statistical tests. All experiments have been randomized using simple randomization. All datasets for this study are included in the manuscript and Table S1.

## Results

### Morphology and anatomy of fully turgescent *R*. *myconi* leaves

In general, *R. myconi* plants possess a rosette-like growth habit consisting of larger leaves at peripheral and smaller, slightly twisted ones in the center of the rosette (Figure 1A). Each plant develops several cylindrical inflorescence stalks carrying one or two violet flowers. The thick and hairy leaf blades are typically rhombic in shape and possess a reticulate venation pattern with pronounced secondary veins diverging from the midrib at small angles (Figures 1A,B, Figure S1). Higher order veins are less pronounced. The petioles are typically longer in peripheral than in central leaves (Figure 1A) and possess semi-circular cross-sections (Figure 1C). The cells of these petiolar tissue types are more or less isodiametric in both axial and transverse direction, with the exception of the cells of the vascular tissues, which are longitudinally elongated (Figures 1C,D, Figure S2). Lignified vascular tissues are easily distinguishable from other tissue types after TB (dark-blue), AO (bright-yellow), or Phloroglucin-HCl (red) staining (Figures 1C,D, Figure S3), as well as due to their individual arrangement. Generally, all vascular tissues are arranged in parallel orientation and close to the abaxial side of the petiole. Depending of the point of sectioning, each cross-section shows a large arc-shaped vascular complex (consisting of several fused vascular strands) at a central abaxial location, as well as, two or more isolated vascular strands close to its edges (Figure 1C). Moreover, longitudinal petiolar thin sections reveal spiral thickenings inside the xylem vessels (Figure 1D). In-between the epidermal and vascular tissues, *R. myconi* petioles have small parenchymatous cells (located abaxially and densely packed) and large parenchymatous cells with regularly interspersed lacunae (located adaxially and loosely packed), as well as, cells of varying shapes and sizes within a distinct transition zone (mainly surrounding the large vascular complex) forming gradients between regions of cells with wide and narrow diameters (Figure 1C). Finally, the tissue arrangement of lamina is typical for dicotyledons, comprising both palisaded and spongy parenchyma cells, which are interwoven by the abaxially located leaf veins and midrib (Figure S3). Interestingly, the lamina is wavy and possesses a complex adaxial texture originating from the leaf's abaxial venation pattern (Figure 1B, Figure S1).

### Kinematical, morphological, anatomical, and mechanical alterations during DRE

**Figure 2:**
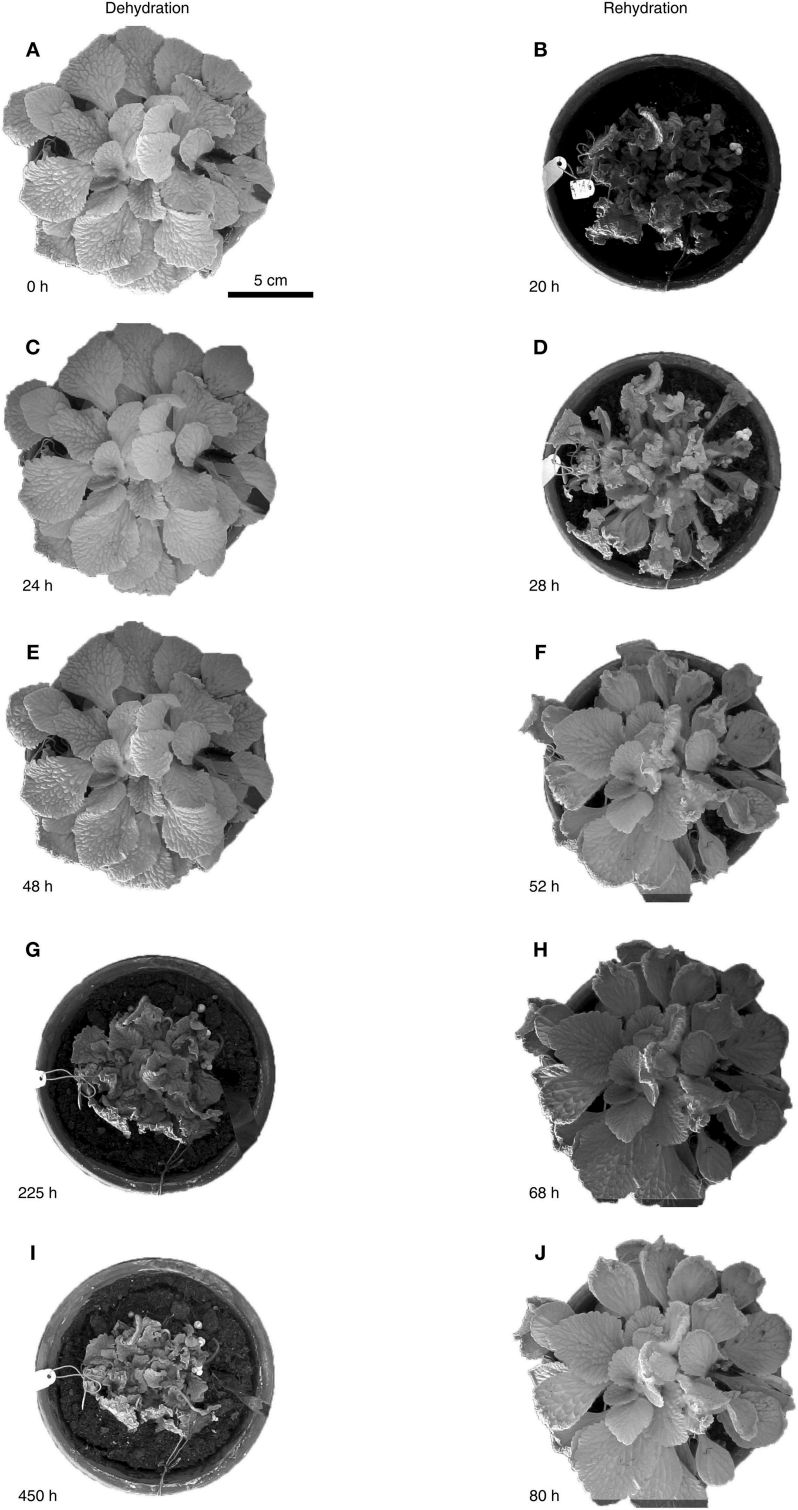
Water status dependent folding kinematics of *R. myconi*. Folding and shrinking of *R. myconi* plants at 0 h **(A)**, after 24 h **(C)**, 48 h **(E)**, 225 h **(G)**, and 450 h **(I)** of dehydration. Unfolding and swelling of *R. myconi* plants after 20 h **(B)**, 28 h **(D)**, 52 h **(F)**, 68 h **(H)**, and 80 h **(J)** of rehydration. *R. myconi* is able to withstand severe drought stress and re-establishes its normal shape upon rehydration. For reasons of simplification the graph depicts the results of one representative experiment.

We evaluated the leaf folding kinematics of whole *R. myconi* plants during a 20-days dehydration and subsequent 4-days rehydration test (Figure 2 and Video S1). Fully turgescent leaves appeared hydraulically pressurized and consequently as stiff. While central leaves were positioned upright, peripheral leaves were positioned parallel to the soil with their adaxial laminae facing upwards (Figures 2A,J). During dehydration the leaf laminae first began to slightly bend downwards (Figures 2C,E). Then, the petioles contracted and moved all leaves toward the center of the rosette, while at the same time the petioles of peripheral leaves gradually bent toward their adaxial sides, thereby enclosing centrally located leaves (Figures 2C,E,G,I). Continued dehydration led to strong shrinkage and folding of the leaf laminae, which also reversed the initially observed downward bending (Figures 2G,I). This complex folding and shrinking behavior of *R. myconi* leaves finally led to a compacted habitus with abaxial leaf parts mainly facing outwards and with the majority of adaxial parts being centrally covered by the shriveled plant. Only after prolonged periods of desiccation (at least 420 h) the leaf color in some cases changed from dark-green to brownish. Contrastingly, increasing water contents quickly expanded the veins of desiccated leaves, which ultimately resulted in petiole elongation and leaf unfolding (Figures 2D,F, Videos S2–S4). Simultaneously, the ongoing rehydration caused an in-plane swelling of the lamina areas situated between the secondary veins (Figures 2D,F). Taken together, these processes fully reversed the previously described dehydration-specific folding kinematics and re-established the initial shapes and positions of all leaves (Figures 2F,H,J).

The alterations of morphometric parameters measured during all DREs show that the petioles of *R. myconi* underwent a uniform 3D shrinkage by simultaneously reducing their lengths, heights and widths by ~ 40% (Table 1). On the contrary, the lengths of the leaf laminae were reduced by ~50%, whereas their widths were only reduced by ~35% (Table 1). Other morphometric parameters did not show notable alterations.

**Table 1:** Leaf morphometric alterations of *R. myconi* from full turgescence to desiccation.

**Morphometry parameters (mm)**	**Median**	**IQR**	**Min**.	**Max**.	***N***	**RWC**
Lamina length	19.2	4.0	13.9	26.3	6	0–10%
Lamina width	16.6	3.7	15.2	20.4	6
Leaf thickness (basal)	2.2	0.8	1.1	2.2	6
Leaf thickness (median)	1.4	0.3	0.9	1.8	6
Leaf thickness (apical)	0.5	0.1	0.5	0.6	6
Vein thickness (1st order)	1.4	0.2	1.0	1.5	6
Thickness between veins	0.5	0.1	0.4	0.7	6
Petiolus length	11.5	1.6	5.7	13.4	6
Petiolus height	1.4	0.5	1.0	2.0	6
Petiolus width	1.8	0.2	1.6	2.1	6
Lamina length	37.4	14.3	15.5	69.0	173	>90–100%
Lamina width	25.4	9.8	11.9	56.9	173
Leaf thickness (basal)	2.0	0.7	0.9	3.7	173
Leaf thickness (median)	1.5	0.5	0.7	3.0	173
Leaf thickness (apical)	0.5	0.2	0.3	1.1	173
Vein thickness (1st order)	1.3	0.4	0.5	2.4	173
Thickness between veins	0.4	0.2	0.1	1.1	173
Petiolus length	19.6	8.6	5.4	39.5	173
Petiolus height	2.2	0.7	1.1	3.4	173
Petiolus width	3.0	1.1	1.2	5.2	173

*All parameters are presented as medians together with interquartile ranges (IQR), sample sizes (N) and minimum (Min.) and maximum (Max.) values*.

**Figure 3:**
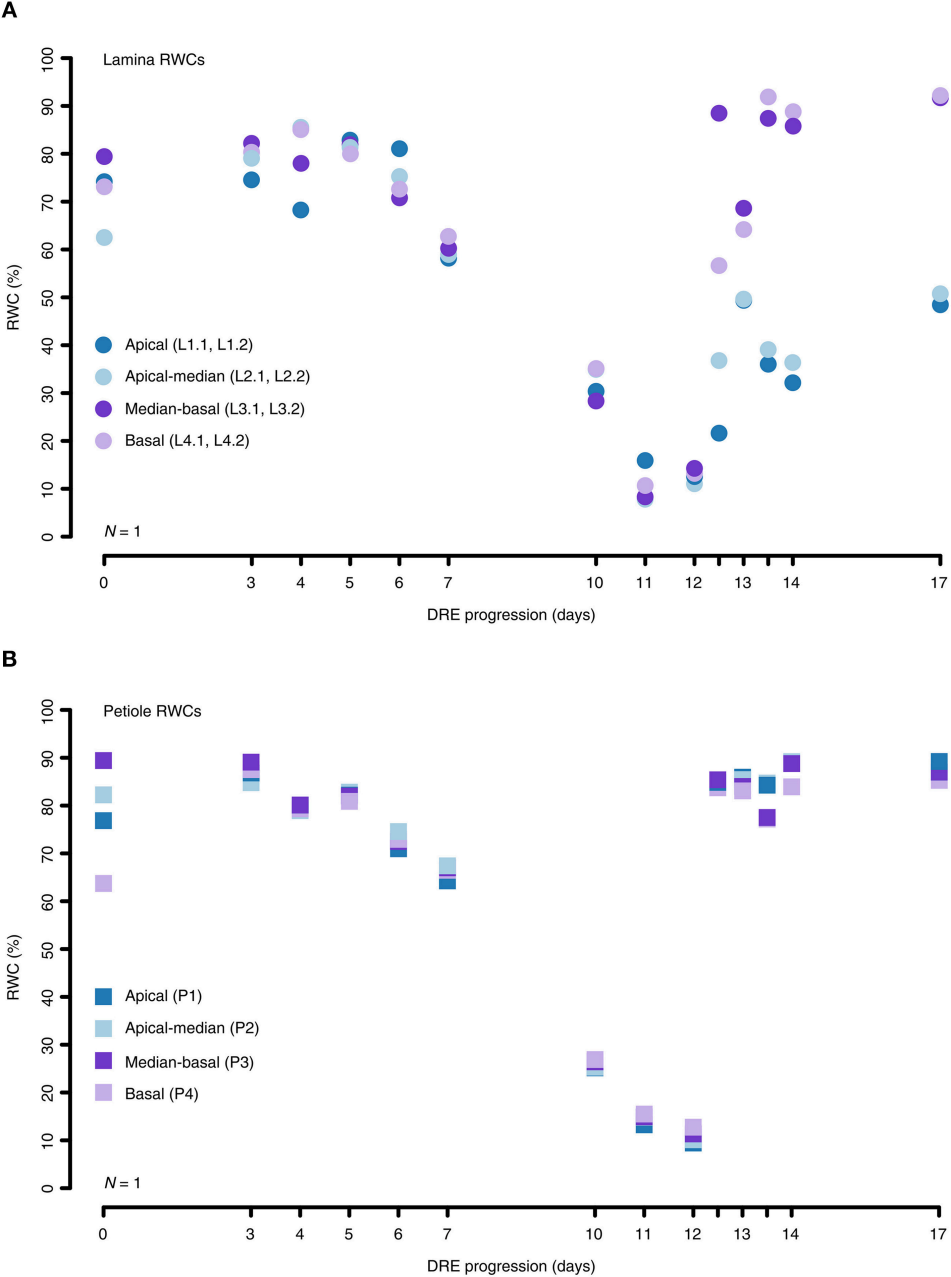
Location-specific RWC alterations of the lamina and petiole of *R. myconi* during DREs. **(A)** Different lamina sampling locations (circles) showed that the local RWCs during dehydration hardly differ. However, sometimes apical lamina parts recovered less than basal lamina parts during rehydration. **(B)** Local RWCs of petiole samples (squares) altered similarly throughout the complete DRE. For reasons of simplification the graph depicts the results of one representative experiment. The origin of each sample is color-coded: blue: apical sampling location; light-blue: apical-to-median sampling location; purple: median-to-basal sampling location; light-purple: basal sampling location. Further information on individual sampling locations is given in parentheses and Figure 1B. Dehydrated samples were collected from day 0 to day 12, whereas progressively rehydrated samples were gathered after last dehydration measurement on day 12.

Furthermore, we performed location-specific RWC measurements to identify water status differences between distinct leaf parts and to monitor their variations throughout a generalized DRE. In general, local RWCs turned out to be very consistent across the whole leaf during dehydration. On average, the RWC values varied by an interquartile range (IQR) of ~5% around the median of any dehydration test interval. During rehydration, the amount of variation around the median per test interval increased to an average IQR of ~31%. Moreover, the local RWCs of both the lamina and the petiole samples displayed uniform dehydration patterns showing IQR values below ~5 and ~0.5%, respectively, across all test intervals (Figures 3A,B). On the other hand, the rehydration patterns of the lamina and the petiole samples clearly differ with IQR values of ~14 and ~0.5%, respectively, across all test intervals (Figures 3A,B). In the end, the afore-mentioned differences in local RWC values between dehydration and rehydration periods result from the fact that the apical lamina parts recovered ~41% less than the remaining leaf parts (Figure 3A).

**Figure 4:**
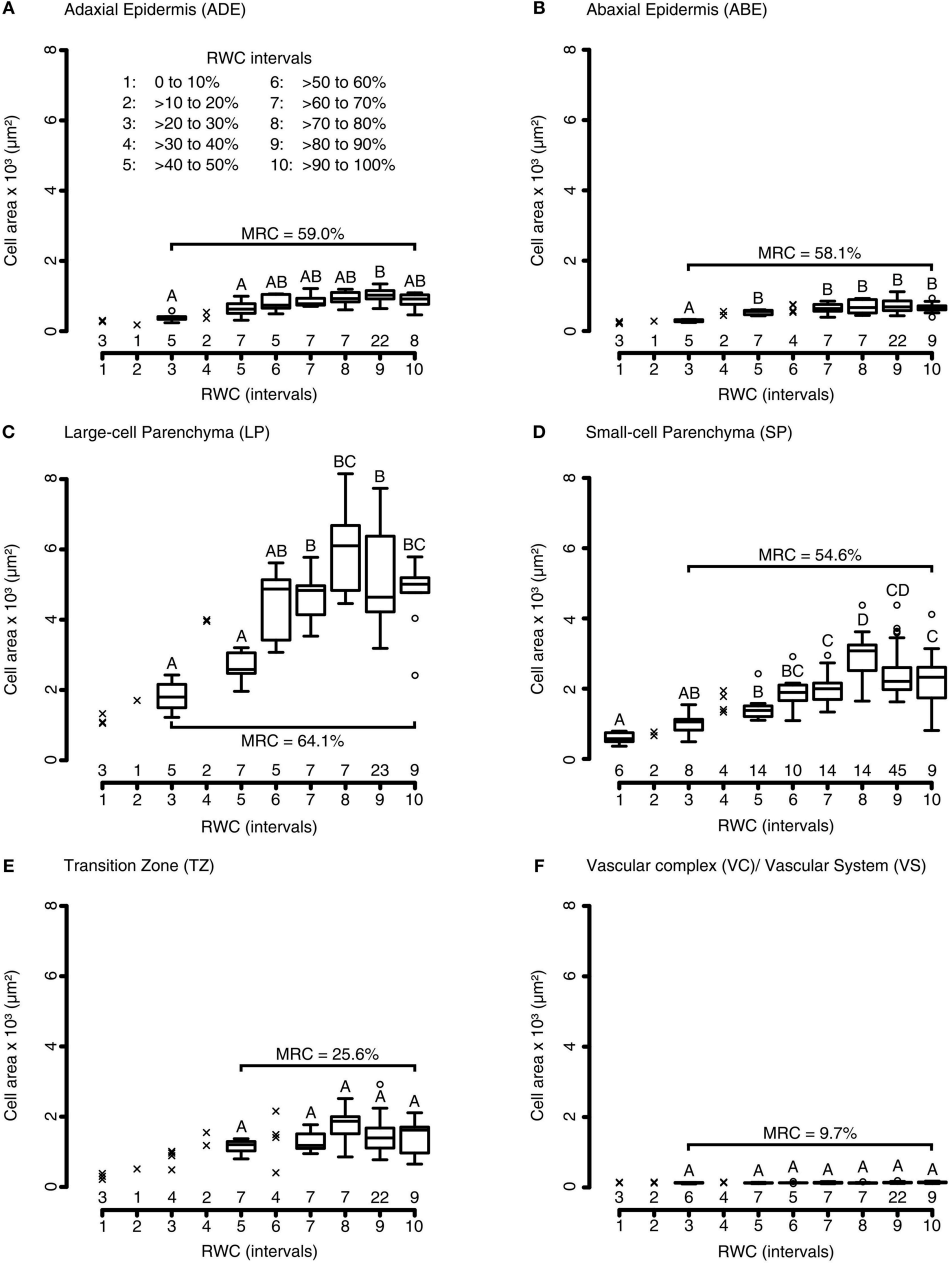
Tissue morphological alterations of *R. myconi* petioles during DREs. Cell area alterations of the adaxial epidermis **(A)**, the abaxial epidermis **(B)**, the large-celled parenchyma **(C)**, the small-celled parenchyma **(D)**, the transition zone between vascular and parenchymatous tissues **(E)**, and the vascular system **(F)** in response to varying RWCs [100 to 0% RWC at 10% intervals, see legend in **(A)**]. The group sample sizes (below) and statistical references (above, Kruskal-Wallis test) are given for each boxplot. Outliers are presented as circles, single values as crosses. The median relative cell area changes (parentheses) are calculated from the cell areas at full turgescence (RWC interval 10) and those at the lowest RWC interval with *N* > 5 (usually interval 3, only for TZ interval 5 was used). ABE, abaxial epidermis; ADE, adaxial epidermis; LP, large-celled parenchyma; MRC, median relative cell area change; SP, small-celled parenchyma; TZ, transition zone between the vascular and the surrounding tissues; VC, vascular complex; VS, vascular system.

To quantify the influence of water availability on the petiolar tissue anatomy, we analyzed the tissue-specific cell area alterations of petiolar cross-sections during a generalized DRE. Both parenchymatous and epidermal tissues displayed significant alterations of cell area with median relative changes between 54 and 64%. Testing for significant differences between cross-sectional cell area and RWC values at given test intervals, the Kruskal-Wallis analyses give the following results for the adaxial epidermis: *X*2 (6) = 23.61, *P* = 6.17·10^−4^; for the abaxial epidermis: *X*2 (5) = 19.23, *P* = 1.74·10^−3^; for the small-celled parenchyma: *X*2 (7) = 68.38, *P* = 3.13·10^−12^; and for the large-celled parenchyma: *X*2 (6) = 30.57, *P* = 3.06·10^−5^ (pairwise Wilcoxon rank sum *post-hoc* testing) (Figures 4A–D). Whereas, the cell areas of parenchymatous tissues changed instantly, the cells of epidermal tissues started to change after a short delay in time (Figures 4A–D). The parenchymatous cells in the transition zone located around the curved vascular complex did only change by 25.6%, which is a non-significant decrease compared to the cell areas measured at full turgescence (Kruskal-Wallis test for transition zone: *X*2 (4) = 6.42, *P* = 0.17; pairwise Wilcoxon rank sum *post-hoc* testing) (Figure 4E). Not surprisingly, the lignified cells of the vascular complex decreased non-significantly in their cross-sectional area (median relative change: 9.7%) (Kruskal-Wallis test for cells of the vascular system: *X*2 (6) = 6.45, *P* = 0.38; pairwise Wilcoxon rank sum *post-hoc* testing) (Figure 4F).

**Figure 5:**
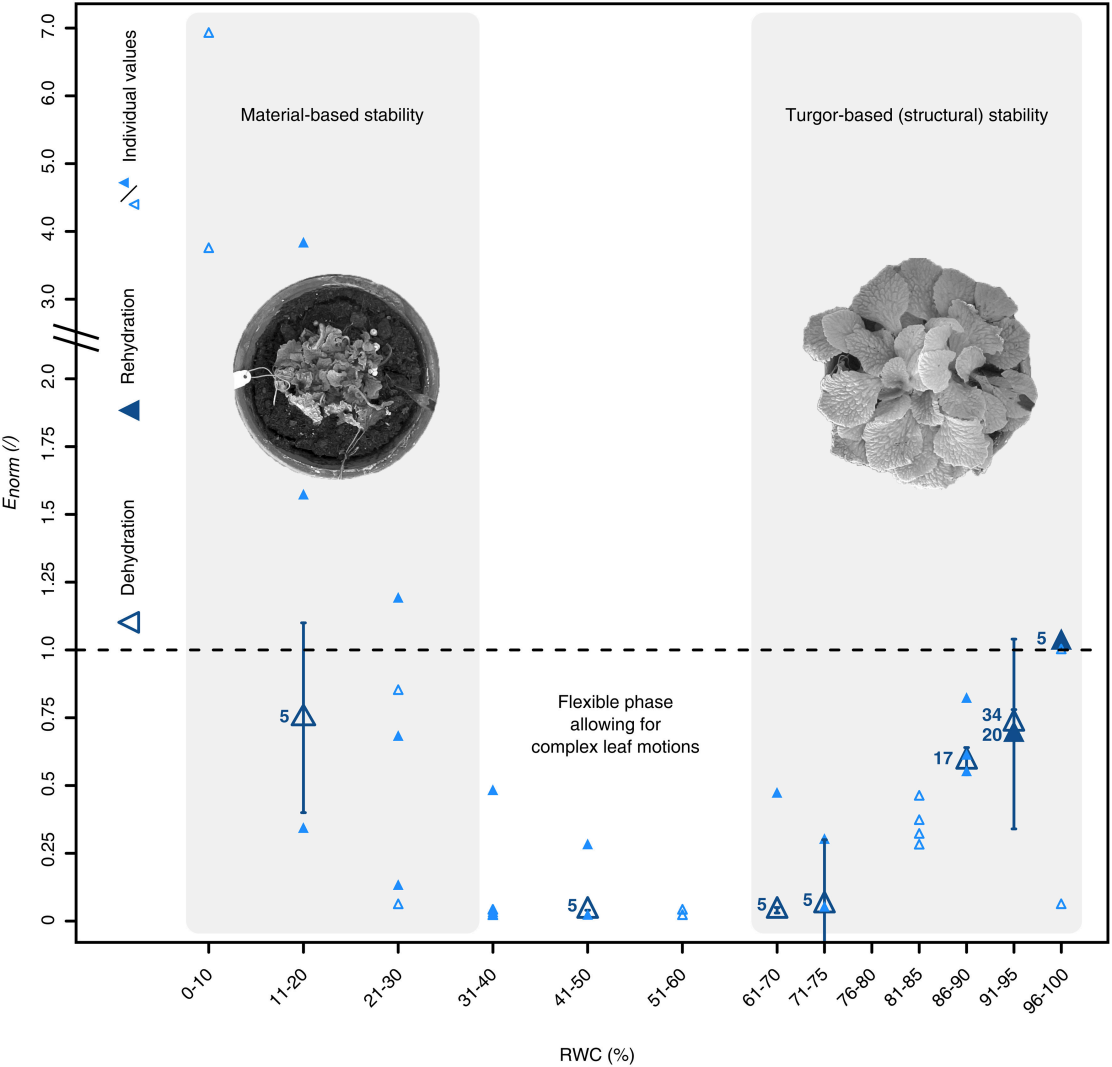
Water status dependent alterations of the petiolar biomechanics of *R. myconi*. Dehydration (open triangles) induces a triphasic mechanical behavior, which is characterized by a turgor-based structural stability at high and a material-based stability at low water contents, and is fully reversible upon rehydration (closed triangles). The structural bending elastic moduli (*E*) have been normalized to the median bending modulus at RWCs > 90%. The normalized bending elastic moduli (*E*_*norm*_) of each RWC interval are either given as median ± standard error of the median (*N* ≥ 5, large dark-blue symbols), or as individual values (*N* < 5, small light-blue symbols).

Finally, we found that varying water availabilities did not only significantly influence anatomical characteristics of the leaf tissues, but also the plant's mechanical stability (Kruskal-Wallis test, *X*2 (7) = 49.82, *P* = 1.57·10^−8^; pairwise Wilcoxon rank sum *post-hoc* testing) (Figure 5). For each RWC interval, the structural bending moduli (*E*_*norm*_), which have been normalized to the median bending modulus at RWCs > 90%, showed a triphasic behavior during the DRE consisting of a turgor-based structural stability at high (Phase 1), a flexible mechanically state at intermediate (Phase 2) and a material-based stability at low water contents (Phase 3) (Figure 5). Starting at full hydration, *E*_*norm*_ decreased with decreasing RWC values until they reached their minimum at a RWC of ~40–50%. A further RWC decrease led to increasing values of *E*_*norm*_, which exceeded the respective values at full turgescence at RWCs below 20% (Figure 5).

### Evaluation of structural adaptations of *R. myconi* to desiccation

**Figure 6:**
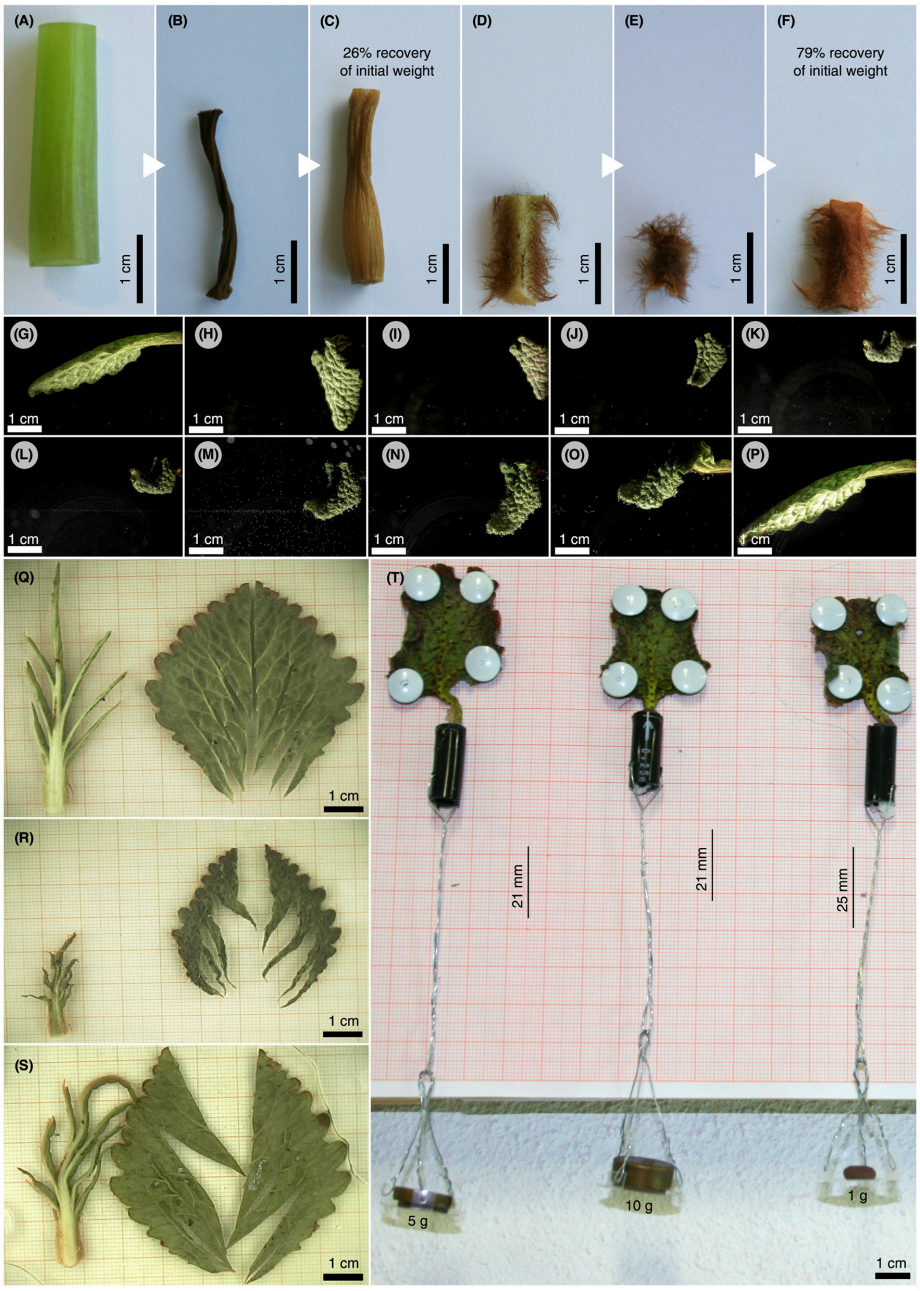
Assessment of structural adaptations to desiccation in *R. myconi*. **(A–F)** Comparative drying experiment using hypocotyl sections of desiccation-intolerant *M. horsfieldii* **(A–C)** and petiolar sections of desiccation-tolerant *R. myconi* **(D–F)** (both Gesneriaceae). Samples were imaged directly after sampling **(A,D)**, after drying **(B,E)** and after rehydration **(C,F)**. Hypocotyl and petiole segments regained 26 and 79% of their initial weights, respectively. **(G–P)** Image sequence illustrating the complex folding and shrinking behavior of a cut-off *R. myconi* leaf during dehydration **(G–K)**, as well as, its recovery after submersion **(L–P)**. The complex 3D folding-unfolding processes are structurally programmed into the leaf. **(Q–S)** Separated lamina and venation system parts, whose spatially complex folding motions were restricted to a 2D plane by clamping between two petri dishes, are shown in the hydrated state **(Q)**, in the strongly contracted dehydrated state **(R)**, and in the rehydrated state **(S)**. The lamina is presumably responsible for (most of) the shrinkage of the leaf, whereas the first order veins are likely to channel the folding-unfolding movements. **(T)** During drying, the petioles exert contraction-induced forces high enough to pull weights.

Comparative manipulation experiments were conducted with cut-off petiole samples of *R. myconi* and cut-off hypocotyl samples of the less tolerant *M. horsfieldii* (also Gesneriaceae). After severe dehydration, the petiole samples of *R. myconi* were able to regenerate about 79% of their initial mass by passive swelling (their initial posture seems also to be fully recovered), whereas *M. horsfieldii* hypocotyl parts could only recover about 26% of their initial mass (Figures 6A–F). Moreover, cut-off whole *R. myconi* leaves showed exactly the same folding and shrinking, as well as, unfolding and swelling kinematics after air-drying and rehydration as described for the leaves of intact plants (Figures 2, **6G–P** and Video S2). These shape changes were fully repeatable as observed in one leaf during three consecutive dehydration-rehydration cycles, which lasted more than 21 days (see Video S3). Furthermore, another DRE with a cut-off leaf with all lamina parts being excised revealed similar folding kinematics upon dehydration as observed in whole cut-off leaves (see Video S4). Additionally, if the lamina parts were separated from the venation system and the separated leaf parts were clamped between two transparent petri dishes (to restrict their 3D folding motions to a 2D plane), a uniform and reversible shrinkage of the leaf lamina between 40 and 50% of its initial area, as well as, a pronounced axial contraction of the secondary veins and the petiole by ~60% could be observed (Figures 6Q–S and Video S5). Finally, the water-loss induced contraction forces of three cut-off petioles, which were pinned to vertically oriented wood planks and stressed by different gravitational loads, were analyzed. During dehydration, the petioles shrank considerably and “pulled” the attached loads upwards. A 1 g mass was pulled upwards by 25 mm, whereas the 5 and 10 g masses were pulled upwards by 21 mm (Figure 6T). Hence, the dehydration-based shrinkage of *R. myconi* petioles generates “pulling forces” of at least 98.1 mN.

## Discussion

Living in shady ravines on northern mountain slopes in the Pyrenees, the herbaceous gesneriad *R. myconi* is bridging long periods of unfavorable environmental conditions, i.e., high irradiance, high temperatures and severe water deficits, similar to its Balkan relatives (Stevanović et al., 1997; Picó and Riba, 2002). Especially, water availability is known as an important environmental factor influencing population performance and geographical plant distribution in Mediterranean regions (Pigott and Pigott, 1993; Dafni and O'Toole, 1994). Therefore, we investigated the influence of water availability on the morphology, anatomy and biomechanics of *R. myconi* with a special focus on structural adaptations to desiccation (i.e., leaf folding kinematics).

How does water availability affect this general morphology of *R. myconi*?—At full hydration the leaves of *R. myconi* were pressurized (turgescent) and stiff, thereby taking up an open posture, in which the plant may efficiently perform photosynthesis (active posture). As soon as the external water availability diminished, the leaf RWCs gradually decreased due to transpiration, which led to decreasing tissue and turgor pressures and manifested in visible leaf drooping during early dehydration. Continued dehydration caused the contraction and upward bending of leaf petioles, as well as, lamina shrinkage, indicating that turgor and tissue pressures had decreased enough to allow for more complex leaf kinematics. Eventually, these dehydration-specific morphological alterations resulted in a protective posture, in which the center of the plant is enclosed by the peripheral leaves, thereby protecting the photosynthetically active tissues and the central vegetation point by minimizing their exposure to external stressors. Similar structural adaptations are also found in other desiccation-tolerant species (Farrant, 2000; Bartels and Salamini, 2001). To date, several studies have demonstrated that both *R. nathaliae* and *R. serbica* were able to recover from RWCs below 10% (Sgherri et al., 2004; Degl'Innocenti et al., 2008). Markovska et al. (1994) further revealed that the recovery of structural changes in *R. serbica* occurs within 2–3 days with water being the basic factor for ultrastructural reconstructions. Here, we demonstrate that *R. myconi* is also able to fully recover from RWC values of < 10% within similar timespans (3–4 days). The rehydration of desiccated *R. myconi* plants led to a rapid increase of their RWC values and therefore to higher turgor and tissue pressures. As a consequence, both the petiole and the leaf veins elongated longitudinally resulting in a partial unfolding of the leaf laminae during early rehydration. Simultaneously, the upward bent peripheral leaves reverted to their initial positions (parallel to the soil surface), thereby exposing the photosynthetically active tissues and the central vegetation point. Thus, most of the afore-mentioned dehydration-specific morphological alterations are fully reversed shortly after rehydration. Finally, the full unfolding of the leaf was achieved by a slow expansion of the lamina.

How are these movements related to the anatomy and the tissue architecture of *R. myconi* leaves?—In general, *R. myconi* petioles possess epidermal, parenchymatous and vascular tissues, which are arranged into a semi-circular cross-section and form an ideal framework for channeling and directing internal forces generated by dehydration or rehydration. The adaxial and abaxial epidermal tissues control the exchange between the plant and its environment and help to protect the inner tissues from external stressors (Robberecht and Caldwell, 1978; Niklas and Paolillo, 1997; Riederer and Schreiber, 2001). Running parallel to the abaxial epidermis, the lignified cells of the vascular system, which both form the isolated peripheral strands and the central arc-shaped complex, provide the long-distance water transport and the mechanical support needed in a terrestrial habitat (Aloni, 1987; Boudet, 2000; Sperry, 2003). Moreover, both the small and large parenchymatous cells presumably contribute to the general structural stability of *R. myconi* under fully turgescent conditions, in addition to their storage function for water, minerals and nutrients (Speck and Vogellehner, 1988; Niklas, 1989, 1992; Spatz et al., 1998; Speck et al., 1998; Rowe and Speck, 2004). Finally, gradual transitions between the afore-mentioned tissue types (especially around the arc-shaped vascular complex) alleviate internal load peaks and shear forces occurring due to marked shrinking and swelling movements caused by water content alterations (Thielen et al., 2013).

Furthermore, DREs revealed that the cell cross-sectional areas of individual tissue types changed differently in response to varying water availabilities. The highest change in cross-sectional area during dehydration and rehydration was visible in the large parenchymatous cells (~64%). Although adaxial (~59%) and abaxial (~58%) epidermal cells displayed a short delay in time before shape alteration in contrast to parenchymatous cells, they underwent similarly high changes in cross-sectional area during DREs. Most likely, this initial epidermal “resistance” is caused by highly adapted cuticle layers and thicker cell walls (Niklas and Paolillo, 1997; Riederer and Schreiber, 2001). Although changing to a lesser extent (~55%), the cross-sectional cell area changes of the small parenchymatous cells were similar to those of the large parenchymatous cells. On the other hand, the cells of the vascular tissues (~10%) and the parenchymatous cells of the transition zone (~26%) did not display significant cell area changes during dehydration and rehydration. However, the latter showed a clear (qualitative) tendency toward smaller cell areas during dehydration. Therefore, the small changes in the cross-sectional cell areas in the transition zone might be an experimental artifact, since the collection of sufficient data at very low water states was not possible for this tissue type. Contrastingly, the lignification of water conduction elements in vascular strands and vascular complexes hindered lateral shrinkage and prevented these cells from significant alterations in cross-sectional area during a DRE (Aloni, 1987; Boudet, 2000; Sperry, 2003).

Taken together, these results unravel the tissue architecture of *R. myconi* leaves at full hydration and at varying water contents. In order to complete the understanding of macroscopic plant movements (i.e., lamina folding, petiole bending or petiole contraction), Niklas (1992) further suggests to analyze the cellulose microfibril arrangement of the involved tissues. When cells absorb/expel water, e.g., in the course of a DRE, it is known from literature that the direction of expansion/shrinkage is mainly perpendicular to the orientation of cellulose microfibrils (Baskin, 2005; Forterre, 2013). For example, collenchymatous cells walls contain longitudinally oriented cellulose microfibrils, which is why they mainly deform radially during dehydration and rehydration (Leroux, 2012). Contrastingly, the walls of full-grown parenchymatous cells typically have no particular microfibril orientation, thereby changing isotropically (Shtein et al., 2016).

As described above, the first noticeable morphological alteration of *R. myconi* in response to drought stress was the contraction of its leaf petioles. This movement results from significant reductions in cell volume simultaneously occurring in the majority of the petiolar tissues during dehydration. Here, the maximum absolute shrinkage was observed in the parenchymatous tissues. Consistently, a recent study on the anatomical specificities of *R. serbica* and *R. nathaliae* discussed the potential supportive role of parenchymatous cells in axial organ contraction (Rakić et al., 2017). In particular, due to their direct connection to neighboring vascular cells via cell-cell junctions combined with a more pronounced reaction to changes in water availability, parenchymatous cells are thought to passively generate mechanical stresses. In order to achieve the observed contraction of the leaf petiole, these mechanical stresses need to be sufficiently high to longitudinally compress and/or bend xylem vessels in the veins and the midrib (at least to some degree), which may be facilitated by their spiral thickenings (Schiller et al., 1999; Rakić et al., 2017). More or less parallel to petiole contraction, a second dehydration-induced leaf movement could be observed, the upward bending of the leaf petioles. Here, the shrinkage due to water loss is likely to occur faster in thin-walled parenchymatous cells than in the thicker lignified cells of the curved vascular bundle complex. Thus, the leaf petioles of *R. myconi* act as a hygroscopic functional bi-layer comprising both an actively deforming adaxial layer (mainly low-density parenchyma) and a resistive abaxial layer. The resistive nature of the abaxial layer mainly originates from the dense and lignified vascular tissues (both the strands and the complex). Additionally, the asymmetry in cell density between adaxial and abaxial parenchymatous tissues is thought to dictate the direction of bending toward the less dense adaxial side, as described for *Selaginella lepidophylla* by Rafsanjani et al. (2015). Thirdly, the slowest dehydration-induced movement is the complex folding of the leaf lamina. Due to their high degree of isodiametry and the random orientation of their microfibrils, the parenchymatous cells within the leaf lamina are generally thought to shrink/swell isotropically. However, morphometric analyses indicate that the laminae displayed stronger shrinkage in axial than in transverse direction. Presumably, the latter phenomenon was an artifact caused by the longitudinal contraction of the leaf's midrib and veins, which superimposed the isotropic volume changes of intercostal lamina parts. Moreover, the complex folding and compaction motions are presumably predefined by the waviness of the leaf blade, as well as, guided by the filigree pattern of the abaxially located venation system.

How are the biomechanical properties of *R. myconi* leaves changing during dehydration and rehydration in order to perform such complex morphological alterations?—The dependence of Young's moduli of herbaceous plant organs and tissues on the turgor pressure has been highlighted by several authors in the past (Falk et al., 1958; Speck and Vogellehner, 1988; Caliaro et al., 2013). For example, Caliaro et al. demonstrated for *Caladium bicolor* “Candyland” that tissue and turgor pressures correlate with the petiole flexural rigidity (Caliaro et al., 2013). Accordingly, turgor and tissue pressure are also thought to be the main contributors to the stability of *R. myconi* in the “green state” (active posture at full hydration). Here, the water-stress induced reduction of RWC values resulted in a drastic decrease of the bending mechanical properties of the petiole, highlighting a turgor-based structural stability between full turgescence and moderate dehydration, i.e., RWCs between 100 and 60% (Phase 1). Between RWC values of 60 and 30%, the decline in mechanical stability was maximal, rendering the leaf material flexible enough to undergo the afore-mentioned morphological alterations (Phase 2). Interestingly, the transition from dehydration, which is the occurrence of steady water loss, to desiccation, which is the final result of dehydration where the water status is equilibrated with the dry air, falls into the same RWC range (Zhang and Bartels, 2018). Concurrent with a RWC decrease from 30 to close to 0%, the bending modulus of *R. myconi* petioles gradually increased again (Phase 3). Presumably, this increase in mechanical stability can be addressed to the mechanical properties of the cell wall polymers, whose significance for the overall petiolar stability increases with ongoing dehydration. Moreover, the measured values represent “bending moduli” of a complex plant organ: the petiole, which is composed of different tissue types each contributing according to its own bending modulus and its axial second moment of area, representing its arrangement in the petiole (Speck et al., 1990; Speck, 1994; Speck and Rowe, 2003; Rowe and Speck, 2004; Rüggeberg et al., 2009; Caliaro et al., 2013). However, it is striking that the bending moduli in this phase exceeded those of the turgor-based (structural) stability phase at full hydration. Nevertheless, this material-based stability kept the desiccated plant in its protective posture throughout the drying period.

Do the presented adaptations to desiccation in the leaves of *R. myconi* also include structural means?—First of all, the manipulation experiments revealed that the passive water uptake after dehydration of excised dried plant parts is approximately three times higher in poikilohydric *R. myconi* compared to its homoiohydric relative *M. horsfieldii*. From an eco-physiological perspective, this can potentially be regarded as an important adaptation, as it enables these plants to passively take up water and to immediately start the “resurrection” from their cryptobiotic state until physiological processes have been re-activated. As intact and cut-off leaves are capable of repetitive leaf folding (as observed in multiple consecutive DREs), the respective actuation principle is presumably of passive nature and enabled by structural adaptations. Moreover, detailed analyses of the leaf-folding behavior of cut-off *R. myconi* leaves revealed that the petiole and the leaf veins mainly channel (in terms of actuation), whereas the lamina fine-tunes (in terms of folding and compaction) the complex 3D leaf motions by swelling and shrinking. Probably, these described phenomena originate from ultrastructural modifications of the cell wall, i.e., cell wall folding (Vicré et al., 1999, 2004; Moore et al., 2006; Farrant et al., 2007). Lastly, the forces generated by passively shrinking petioles were at least 98.1 mN and with that high enough to pull the respective leaf material toward the center of the rosette.

How do these structural and biomechanical traits contribute to the desiccation tolerance of *R. myconi*?—From literature it is known that desiccation-tolerant plant species predominantly possess physiological adaptations, which usually function at cellular level and mainly improve metabolic processes in order to alleviate desiccation-induced cell damages (Markovska et al., 1994; Müller et al., 1997; Stevanović et al., 1997; Farrant, 2000; Sgherri et al., 2004; Živković et al., 2005; Degl'Innocenti et al., 2008; Gechev et al., 2013). Additionally, structural adaptations are important for ensuring the protection of fragile and/or important plant parts. *S. lepidophylla*, for example, curls into a compact, nest-ball shape upon dehydration to limit photoinhibitory and thermal damages in arid environments (Rafsanjani et al., 2015). Similarly, *R. myconi* may enclose centrally located leaves and the vegetation point with peripheral ones through complex folding kinematics in order to protect its growth apex and its photosynthetically-active areas from drought and light stress induced damages, whose formation and impact are reviewed by several authors (Walters et al., 2002; Lüttge et al., 2011). Moreover, the water status related change of biomechanical properties guarantees both sufficient stabilization of the above-ground tissues in the light-receiving active posture at high, and the light-avoiding protective posture at low water contents, as well as, sufficient compliance to reversibly switch between these states at intermediate water availability (i.e., leaf folding/unfolding movements).

In conclusion, the desiccation-tolerant gesneriad *R. myconi* is highly adapted to severe water stresses both utilizing physiological (as concisely summarized in the introduction), as well as, structural traits as shown in this study. Furthermore, it was demonstrated that water availability changes specifically alter the tissue properties, and considerably influence the mechanical stability of *R. myconi* in a triphasic manner. In particular, *R. myconi* becomes more compact and less prone to external stressors due to a minimized exposition and adapts its mechanical stability allowing for reversibly switching between an active posture at high and a protective posture at low water contents. Most likely, these phenomena are actuated by the morphological alterations of the leaf veins and petioles, whereas their fine-tuning originates from the folding and shrinking of the lamina. In general, these passive structural adaptations ensure the protection of fragile and/or physiologically important plant parts (i.e., photosynthetically-active areas, growth apex) throughout periods of prolonged water stress, as well as, the start of the “resurrection” from a cryptobiotic state until important metabolic mechanisms have been restored.

## Author contributions

TK designed the study, collected the data, carried out the data assessment and statistical analyses, and wrote the first draft of the manuscript. SD participated in the data collection and assisted the data analysis. SP participated in the design and coordination of the study, helped to interpret the data, and assisted in drafting the manuscript. TS participated in the design of the study, helped to interpret the data, to draft the manuscript, and coordinated the study. All authors gave final approval for publication.

### Conflict of interest statement

The authors declare that the research was conducted in the absence of any commercial or financial relationships that could be construed as a potential conflict of interest.

## Supplementary material

Video S1 DRE of an intact *R. myconi* plant. The video shows the dehydration (20 days) and rehydration (4 days) of an intact *R. myconi* plant.

Video S2 DRE of a cut-off *R. myconi* leaf. The video shows the dehydration and rehydration specific plant movements of a cut-off leaf.

Video S3 Repeatability of DRE-specific plant movements. The video shows three consecutive DREs of the same cut-off *R. myconi* leaf.

Video S4 DRE-specific leaf vein movements. The video shows the plant movements that are only related to DRE-specific alterations within the leaf's venation system.

Video S5 DRE-specific 2D lamina and petiole movements. The video shows the DRE-specific two-dimensional movements of the leaf lamina separated from its petiole and venation system.

Figure S1 Texture and venation pattern of a fully hydrated *R. myconi* leaf. **(A)** Side view of the leaf. **(B)** Top view of the leaf. The leaf displays a pronounced waviness and a very complex pattern of higher order leaf veins. Additionally, the strong leaf pubescence is clearly visible.

Figure S2 Longitudinal thin sections of the leaf petiole of *R. myconi*. **(A)** Longitudinal cut of a *R. myconi* leaf petiole stained with toluidine-blue. **(B)** Close-up of the thin section shown in A. Except for the elongated lignified vascular tissues, the cells of all tissue types are more or less isodiametric in longitudinal direction.

Figure S3 Additional transverse thin sections of the leaf petiole and the leaf lamina of *R. myconi*. **(A)** Cross-section of a leaf petiole stained with Toluidine-Blue. **(B)** Cross-section of a leaf petiole stained with Phloroglucin-HCl to highlight lignified tissues. **(C)** Close-up of a transversally cut vascular strand of the petiole (Phloroglucin-HCl staining). **(D)** Close-up of a transversally cut vascular complex of the petiole (Phloroglucin-HCl staining). **(E)** Cross-section of a leaf lamina stained with Toluidine-Blue. **(F)** Close-up of a transversally cut leaf lamina (midrib region) stained with Phloroglucin-HCl. **(G)** Close-up of a transversally cut vascular complex of the lamina (Phloroglucin-HCl staining). **(H)** Close-up of a transversally cut vascular strand of the lamina (Phloroglucin-HCl staining).

Table S1 Measured data.

## Abbreviations

AO: acridine-orange
DRE: dehydration-rehydration experiment
DT: desiccation tolerance
DW: dry weight
FW: fresh weight
HDT: homoiochlorophyllous desiccation tolerance
IQR: interquartile range
LEAs: late embryogenesis abundant (proteins)
PDT: poikilochlorophyllous desiccation tolerance
RH: relative humidity
ROI: region of interest
ROS: reactive oxygen species
RWC: relative water content
sHSPs: small heat shock proteins
TB: toluidine-blue
TW: turgescent weight.
