# Supplementary material for: Kinematical, Structural and Mechanical Adaptations to Desiccation in Poikilohydric Ramonda myconi (Gesneriaceae)
Source: Front Plant Sci. 2018 Nov 20;9:1701. doi: 10.3389/fpls.2018.01701 (PMC6256057; doi:10.3389/fpls.2018.01701)
Supplement: Figure S1 — Texture and venation pattern of a fully hydrated R. myconi leaf. (A) Side view of the leaf. (B) Top view of the leaf. The leaf displays a pronounced waviness and a very complex pattern of higher order leaf veins. Additionally, the strong leaf pubescence is clearly visible. [file Data_Sheet_1.PDF]

Supplementary Material S6 – Additional figures

Kinematical, structural and mechanical adaptations to desiccation in poikilohydric *Ramonda myconi* (Gesneriaceae)

Tim Kampowski,[1,2*] Sven Demandt,[1] Simon Poppinga,[1,2] Thomas Speck[1,2]

[1]Plant Biomechanics Group Freiburg (PBG), Botanic Garden, University of Freiburg, Freiburg im Breisgau, Germany.

[2]Freiburg Materials Research Center (FMF), University of Freiburg, Freiburg im Breisgau, Germany.

***Correspondence:**
Tim Kampowski
tim.kampowski@biologie.uni-freiburg.de
ORCID: 0000-0003-3937-4192
Tel: +49-761-2032781

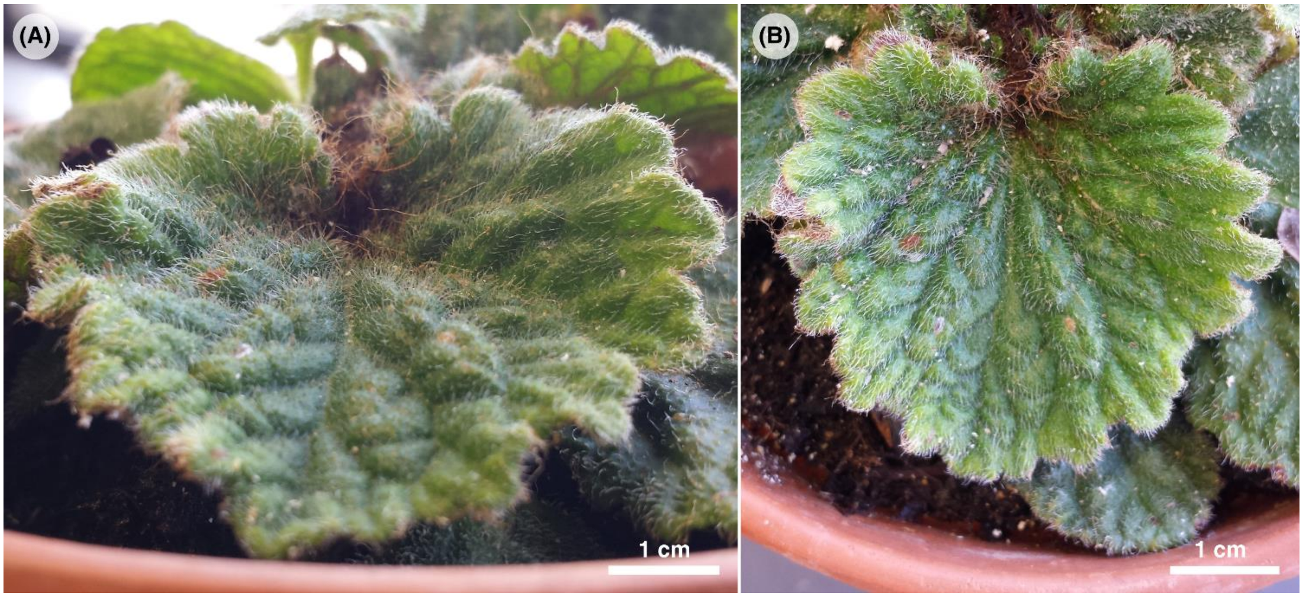

Figure S1. Texture and venation pattern of a fully hydrated *R. myconi* leaf. **(A)** Side view of the leaf. **(B)** Top view of the leaf. The leaf displays a pronounced waviness and a very complex pattern of higher order leaf veins. Additionally, the strong leaf pubescence is clearly visible.

This is a provisional file, not the final typeset article

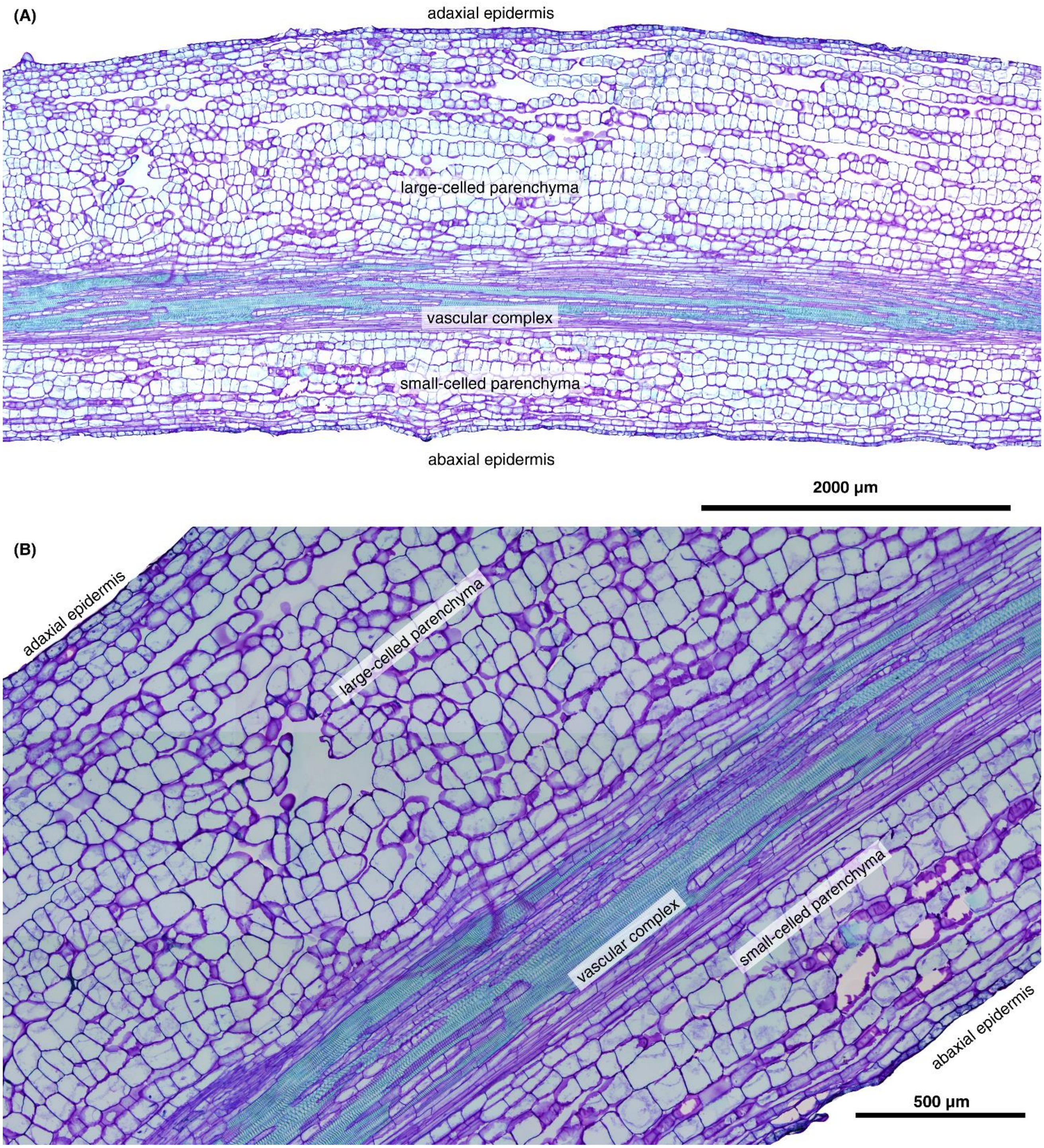

Figure S2. Longitudinal thin sections of the leaf petiole of *R. myconi*. **(A)** Longitudinal cut of a *R. myconi* leaf petiole stained with toluidine-blue. **(B)** Close-up of the thin section shown in A. Except for the elongated lignified vascular tissues, the cells of all tissue types are more or less isodiametric in longitudinal direction.

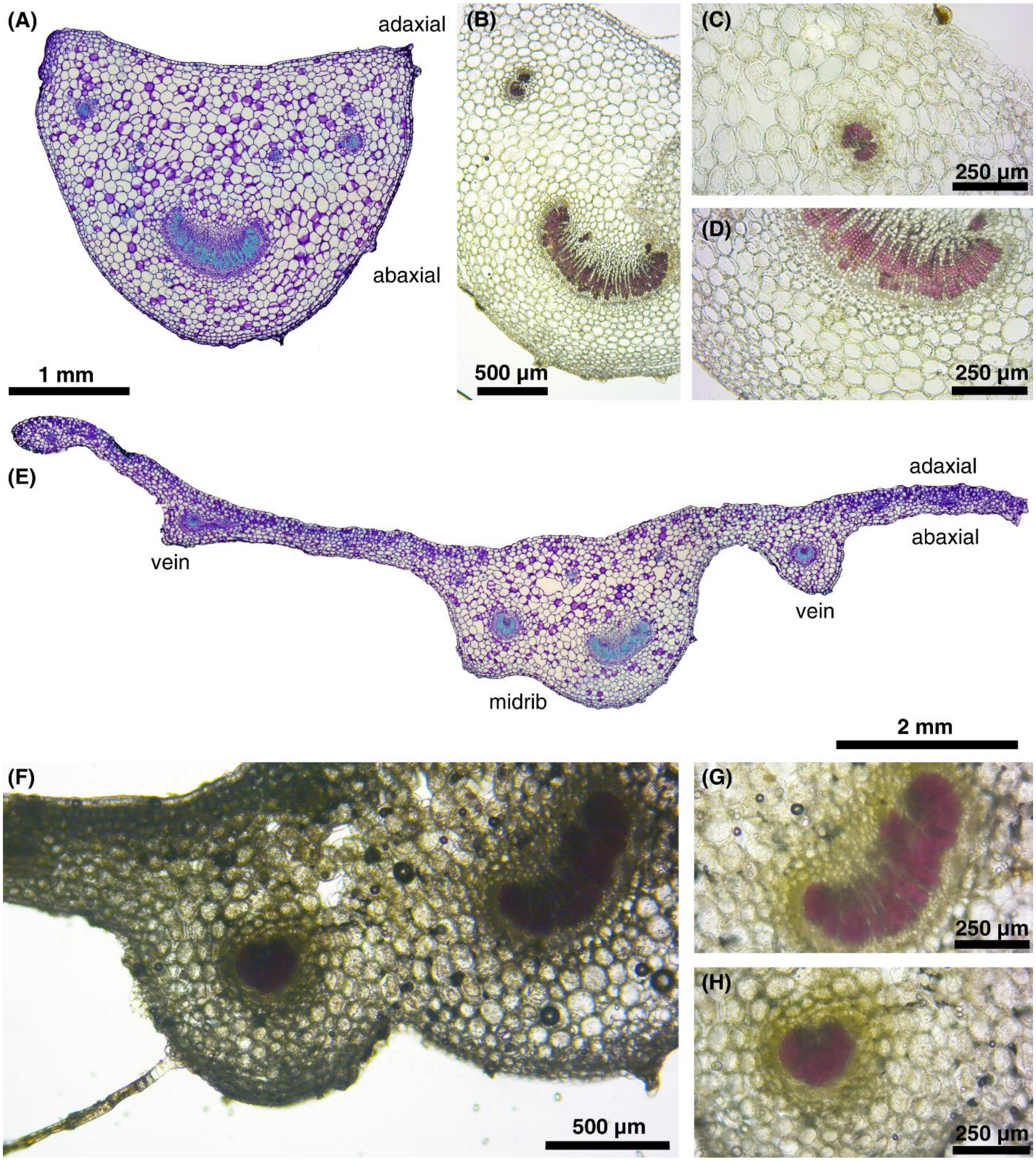

Figure S3. Additional transverse thin sections of the leaf petiole and the leaf lamina of *R. myconi*. **(A)** Cross-section of a leaf petiole stained with Toluidine-Blue. **(B)** Cross-section of a leaf petiole stained with Phloroglucin-HCl to highlight lignified tissues. **(C)** Close-up of a transversally cut vascular strand of the petiole (Phloroglucin-HCl staining). **(D)** Close-up of a transversally cut vascular complex of the petiole (Phloroglucin-HCl staining). **(E)** Cross-section of a leaf lamina stained with Toluidine-Blue. **(F)** Close-up of a transversally cut leaf lamina (midrib region) stained with Phloroglucin-HCl. **(G)** Close-up of a transversally cut vascular complex of the lamina (Phloroglucin-HCl staining). **(H)** Close-up of a transversally cut vascular strand of the lamina (Phloroglucin-HCl staining).

This is a provisional file, not the final typeset article
